# Periapical Granuloma: A Case Report

**DOI:** 10.7759/cureus.116288

**Published:** 2026-09-15

**Authors:** Karthik Rajaram Mohan, Ramachandra Reddy Gowda Venkatesha, Saramma Mathew Fenn, Reethika Rathan, Kumar Appusamy

**Affiliations:** 1 Oral Medicine and Radiology, Vinayaka Mission's Sankarachariyar Dental College, Vinayaka Mission's Research Foundation (DU), Salem, IND

**Keywords:** granuloma, histiocytes, mandibular molar, periapical granuloma, lymphocytes

## Abstract

A periapical granuloma is a fleshy mass of tissue invaded by varied numbers of chronic inflammatory cells, namely, epitheloid cells (modified tissue macrophages), mast cells, lymphocytes, and plasma cells, usually attached to the apex of a nonvital carious tooth as a sequelae of periapical infection from the diseased pulp. The growth of periapical granulomas develops in spurts amidst recurrent acute exacerbations. Periapical granulomas may form as the initial periapical pathosis or as a result of a quiescent periapical abscess. These lesions might not always be static. Radiographic changes are absent in the early phases of the disease. We reviewed the numerous immunological expressions, inflammatory mediators that endorse periapical granuloma progression, and prognostic markers.

## Introduction

A mass of persistently or subacutely inflamed granulation tissue at the apex of a nonvital carious tooth is referred to as a periapical granuloma. The lesion does not microscopically exhibit true granulomatous inflammation; hence, the nomenclature that is most usually used is not completely true [1]. The more accurate word is "apical periodontitis," albeit the clinician may find it perplexing [1]. Neutrophils predominate in the early phases of infection, and radiographic changes are absent [1]. The term "acute periapical periodontitis" refers to this stage [1]. During the acute phase of inflammation, neutrophils are the main inflammatory cells involved, and they release prostaglandins, which trigger osteoclasts to resorb the surrounding bone and cause a periapical radiolucency that may be detected [1]. According to researchers, this bone deterioration is an effort to stop the spread of infection and make room for the entry of defense cells trained to combat the infectious process [1]. Chronic inflammatory cells like lymphocytes gradually take control of the host response and secrete mediators that inhibit further osteoclast activation while simultaneously promoting fibroblast and microvascular activity [1]. Because of these processes, persistent lesions frequently show no symptoms and minimal additional radiographic change [1]. With a predominant histiocytes (macrophages in connective tissue) and multinucleated large cells, this disease differs from granulomatous inflammation. In addition to chronic inflammatory cells such as lymphocytes and granulomatous inflammatory tissue, there is significant neutrophilic infiltration during an acute exacerbation of a periapical granuloma [2].

## Case presentation

A 37-year-old female reported a chief complaint of pain in her right lower back tooth region for the past six months. Her vitals were stable, and her medical history revealed no systemic disorders. An intraoral examination revealed a carious right mandibular second molar. Three-dimensional and sequential multiplanar sectional images of her right mandibular posterior region were obtained using a Carestream CS9600 CBCT machine with a field of volume of 12 x 10 cm and exposure parameters of 120 KVp, 5 mA, 12 secs, and 796 mGycm^2^. Cone-beam computed tomography (CBCT) uses a cone-shaped X-ray beam to create three-dimensional images of the teeth, jawbone, and surrounding structures. It consists of a gantry in which the X-ray source and detector rotate synchronously in a fixed fulcrum around the patient. The CBCT scan simultaneously creates multiple images in three orthogonal planes: coronal, sagittal, and axial sections. These sectional images in the CBCT aid in diagnosing and determining the exact extent of periapical pathologies around the teeth. CBCT revealed a 7.60 mm x 5.58 mm well-defined radiolucency in the coronal section and 2.91 mm x 2.28 mm in the axial section with loss of laminadura in the root apex of 47 (Figure 1A-1B).

**Figure 1:**
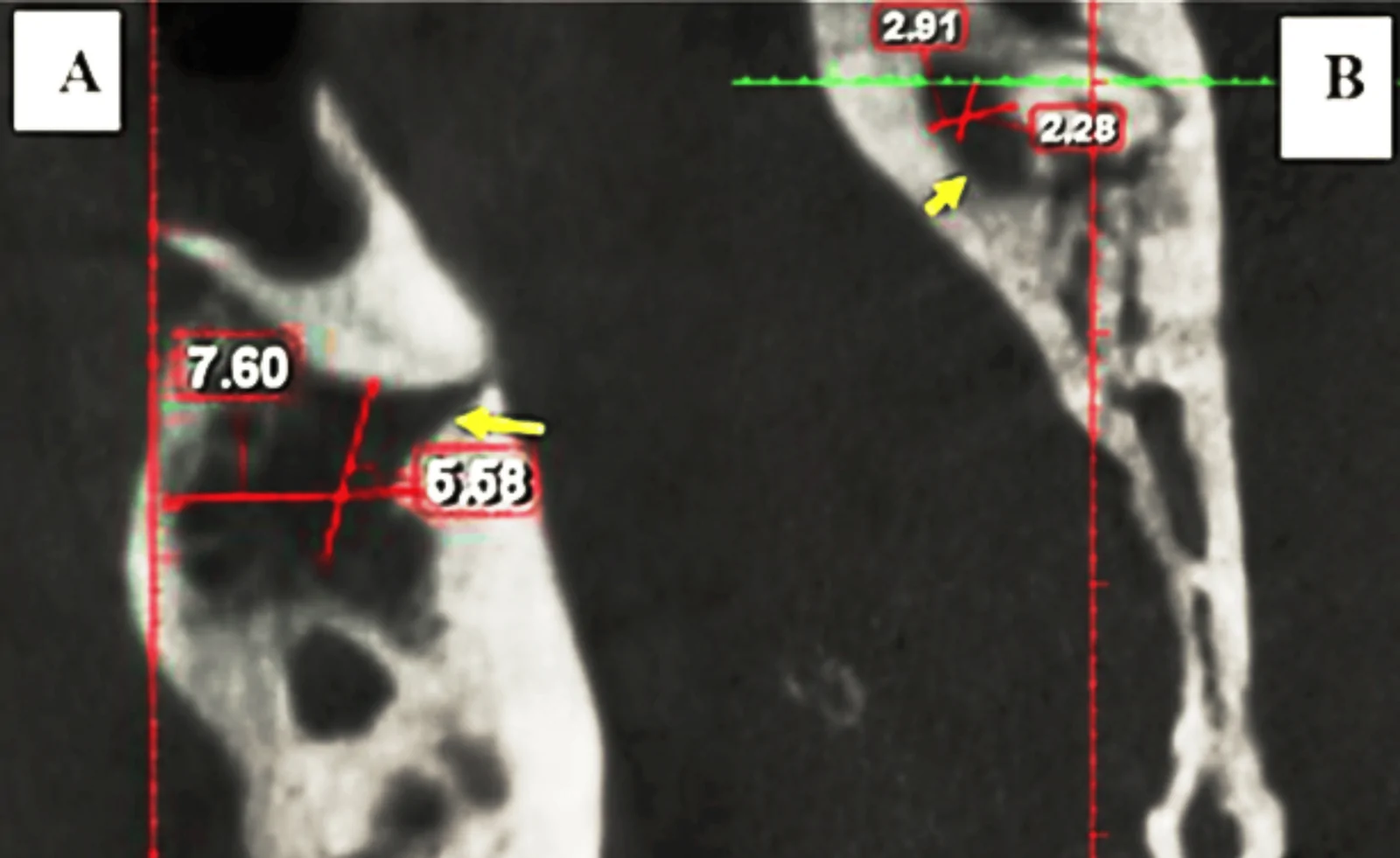
(A) CBCT coronal section revealed a well-defined radiolucency measuring 7.60 mm x 5.58 mm in the periapical root region with loss of lamina dura in the right mandibular second molar. (B) The axial section of the CBCT revealed a well-defined radiolucency measuring 2.91 mm x 2.28 mm in the root apex of the right mandibular second molar CBCT: Cone-beam computed tomography

The differential diagnosis includes a periapical cyst. The periapical cyst typically has a well-defined radiolucency greater than 1.5 cm in diameter and may cause cortical plate expansion, thinning, or perforation. A dental extraction was performed under the inferior alveolar nerve block, and the carious right mandibular second molar was extracted, which revealed a periapical granulomatous tissue in the periapical mesial root tip region (Figure 2).

**Figure 2:**
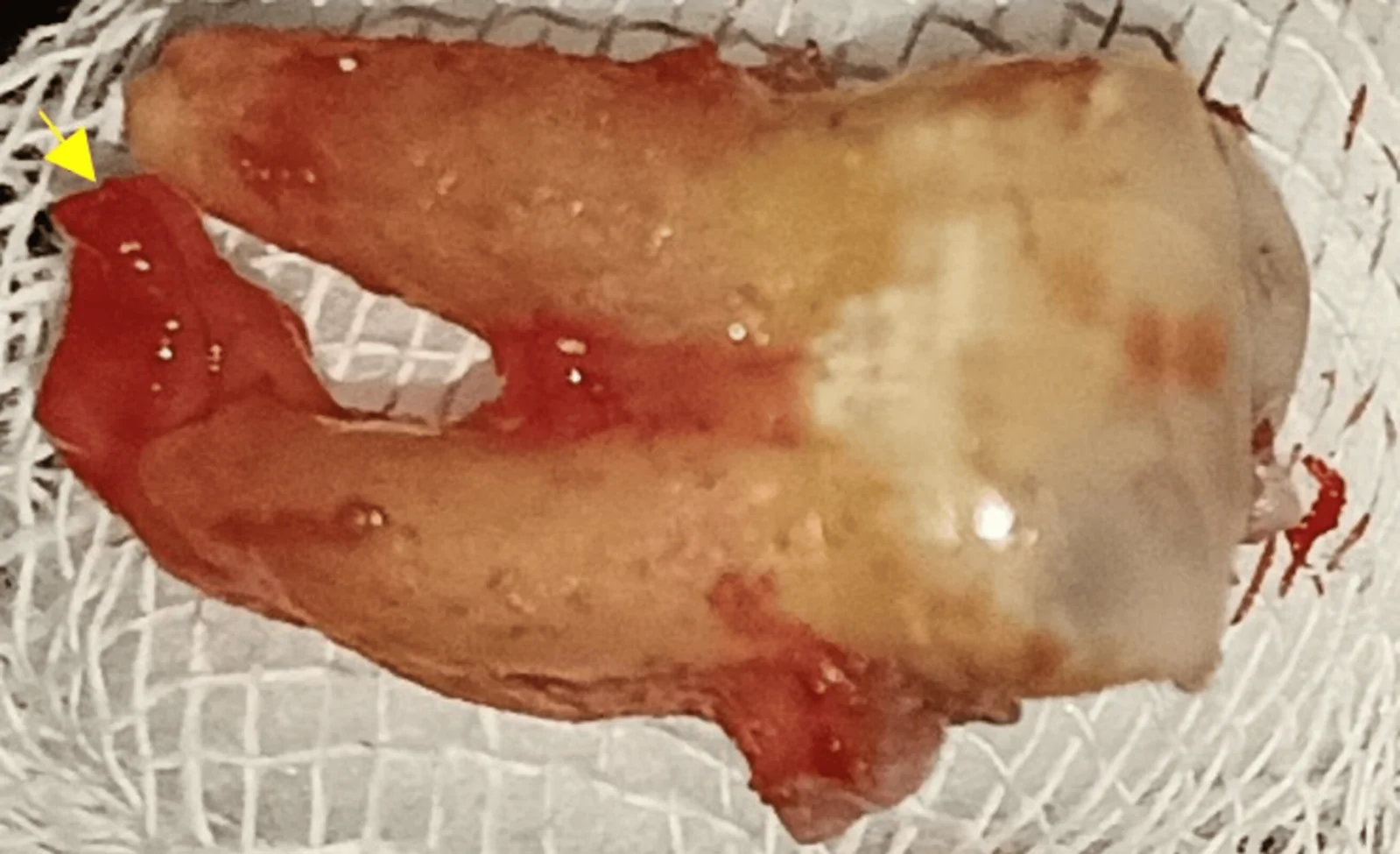
Extracted right mandibular second molar revealed a periapical granuloma (yellow arrow)

## Discussion

Definition

A periapical granuloma is a low-grade infection that occurs as a sequela to pulpitis or acute periapical periodontitis and typically contains a mass of tissue microscopically containing epitheloid cells (modified macrophages) [2].

Classification of periapical granuloma

Based on histological characteristics, periapical granulomas were divided into early, intermediate, and late or healing stages [3]. In the early stage, there is macrophage cell infiltration of the periodontal ligament, inflammatory effusion (exudates), significant macrophage infiltration, frothy (pale) cytoplasm, a few lymphocytes, hyperemia, with or without neutrophils, and edema [3]. There are numerous blood vessels, and other chronic inflammatory cells, such as plasma cells and lymphocytes, predominate in the intermediate stage [3]. The intermediate stage, which includes apical root resorption and the surrounding supporting alveolar bone, is also known as the stage of increased permeability resulting in increased vascularity. In the late or healing stage is typically characterized by fibroblasts, which are larger in size and granules of hemosiderin within a fibrous connective tissue stroma, few chronic inflammatory cells, and blood vessels [3]. A periapical granuloma with fibroblast and endothelial cell development, numerous minute vascular channels, and macrophage, lymphocyte, and plasma cell infiltration are all characteristics of this stage [3].

Histological features

The presence of epithelium is the most significant histological characteristic of periapical granuloma [3]. The epithelium develops from epithelial remnants of Malassez in the periodontal ligament, though it may also come from the maxillary sinus respiratory epithelium when the periapical lesion has penetrated the sinus wall, oral epithelium growing through a fistulous tract, the apical proliferation of oral epithelium from a periodontal pocket, or a bifurcation or trifurcation involvement by periodontal disease, both of which are associated with the formation of periapical granuloma. The epithelium is restricted to the area just next to the periodontal ligament in early periapical granulomas [3]. Over time, inflammatory stimuli cause the epithelium to proliferate in an attempt to ward off the irritation that is discharged from the apical foramen, which enlarges and manifests as anastomosing cords or sheets of cords of stratified squamous epithelial cells [3]. Therefore, if left untreated and with a persistent inflammatory response that stimulates the epithelium, every periapical granuloma has the potential to develop into a periodontal cyst [3]. Rushton bodies are ring-shaped, eosinophilic entities found in the periapical granuloma that resemble hyalinized collagen [3].

Immunological expression and inflammatory mediators in periapical granulomas

Chen et al. explained that Toll-like receptor 2 (TLR2) was strongly expressed by plasma cells and lymphocytes, indicating that these cells play a significant role in the inflammatory process and antigen recognition in refractory periapical granulomas [4]. There were not many dendritic cells that expressed TLR2, indicating that they play a small part in perpetuating these lesions [4]. Ishii et al. stated that round inflammatory cells that express Forkhead box transcription factor class O3a (Foxo3a) include neutrophils, T lymphocytes, and B lymphocytes in periapical granulomas [5]. According to these findings, transcription factors associated with Foxo3a may have contributed to the development of periapical granulomas [5]. Cilmiaty et al. stated that dental caries manifests as an increase in nuclear factor kappa B (NF-kB) expression in the dental granuloma tissue [6]. Tamura et al. stated that after being stimulated with the inflammatory cytokine interleukin-1 (IL-1), S100A4 was expressed, and this enhanced tumor necrosis factor (TNF) expression by autocrine or paracrine action, worsening the inflammatory response in periapical granulomas [7]. Batista et al. stated that versican, phosphorylated epidermal growth factor receptor (pEGFR), and a disintegrin and metalloproteinase with thrombospondin motif 1 (ADAMTS-1) were all expressed in the lesions of periapical granuloma [8]. The intricate interaction between molecules involved in the immunoinflammatory response process lends credence to these persistent periapical lesions [8]. He also stated that the pathogenesis of periapical granulomas is caused by the modification of the versican extracellular matrix (ECM) by ADAMTS-1, which could also result in bioactive fragments that activate EGFR and help with the development, growth, and spread of the disease [8]. Martín-González et al. stated that the adipokine leptin is expressed by human periapical granuloma [9]. Bansal et al. stated that the nature, pathophysiology, and biological activity of periapical granuloma lesions are predicted by the average amount of collagen fibers, their birefringence, and the density of mast cells [10].

Kudo et al. described that inflammatory cell in periapical granulomas co-express silent information regulator 2 homolog 1 (SIRT1) and 8-hydroxy-2′-deoxyguanosine (8-OHdG), and resveratrol increases U-937 cell proliferation and stimulates SIRT1 expression [11]. Therefore, he concluded that SIRT1 is expressed by macrophages and that the consequent decrease in oxidative stress promotes the repair of periapical granulomas [11]. Farias et al. stated that the expression of aldehyde dehydrogenase 1 (ALD-1), which has a stem cell-like profile, helps in the healing or repair of periapical granuloma [12].

Diagnostic aids for periapical granuloma

Conventional radiographic techniques, such as intraoral periapical and occlusal radiographs, and specialized radiographic imaging modalities, such as CBCT, aid in the diagnosis of periapical granuloma. According to De Rosa et al., CBCT can be used to distinguish between periapical granulomas and periapical cysts and help analyze their surface textural characteristics [13].

Treatment

Periapical granuloma is treated by either extracting the affected tooth or by root canal therapy, either with or without apicoectomy [13]. The nonsurgical approach to treating periapical lesions involves the use of mineral trioxide aggregate, simvastatin [14,15]. The Niigata University School of Dentistry Cariology Research Unit developed the "Lesion Sterilization and Tissue Repair (LSTR)" therapy, a triple antibiotic paste containing "ciprofloxacin, metronidazole, and minocycline," to disinfect root canals in periapical lesions [15].

Prognostic marker for periapical granuloma

Early detection of this overexpression of angiogenesis factor angiopoietin (ANGPT) and cell-adhesion molecule integrin subunit alpha 5 (ITGA5) expression and correlation in a periapical granuloma tissue sample may indicate the prognosis of periapical wound healing after surgical endodontic treatment [16].

## Conclusions

Periapical granulomas, which occur in the periapical region of the root tip of a carious tooth, present as fleshy tissue containing epitheloid cells, mast cells, and lymphocytes in the root apex of a long-standing carious tooth. The diagnosis of periapical granuloma primarily relies on the radiographic examination. Conventional imaging techniques such as intraoral periapical radiograph, occlusal radiograph, and recently CBCT are invaluable in diagnosing periapical granulomas. Clinically, periapical granuloma often remains asymptomatic or silent, becoming visible only after dental extraction. A comprehensive understanding of periapical granuloma is crucial for dentists, empowering them to make informed decisions about prognosis and treatment.
